# The use of hyaluronic acid based dressings to treat burns: A review

**DOI:** 10.4103/2321-3868.142398

**Published:** 2014-10-25

**Authors:** Cristina Longinotti

**Affiliations:** Research and Development, Anika Therapeutics S.r.l, via Ponte della Fabbrica 3b, 35031 Abano Terme, Italy

**Keywords:** Burns, hyaluronic acid, wound healing

## Abstract

Deep cutaneous lesions such as burns, traumas or ulcers are all conditions characterized by a massive loss of dermis, bringing several important consequences. For the treatment of these conditions, the evolution of material science has made available new dressings based on natural and synthetic polymers. Hyaluronic acid (HA) is involved in many steps of the wound healing process, such as inflammation, granulation and re-epithelialization. In order to overcome the poor physical properties of the native polymer, such as solubility and rapid degradation, insoluble molecules starting from the natural compound were produced via esterification. Thanks to their improved structural properties, the dressings based on these hyaluronic acid derivatives represent a valuable option for the treatment of deep burns. This narrative monograph describes the development and the outcome of the use of these products in burns. The currently available clinical experience suggests that these HA medical devices represent a safe therapeutic method useful for the treatment of acute wounds.

## Introduction

Deep cutaneous acute lesions, such as burns, traumas or ulcers, are all conditions characterized by a massive loss of dermis; therefore the restoration of a full-thickness dermal layer is of primary importance to start the healing process. Once a proper dermal bed has been well developed, wound closure is facilitated spontaneously or by applying a skin graft. Nevertheless, immediate coverage with autologous skin is not always available or possible. In these cases, alternative solutions may be taken into considerations to favor an adequate repair and effective functional recovery of the burned area. Hyalosafe and Hyalomatrix (Anika Therapeutics s.r.l., Abano Terme, Italy) are medical devices based on hyaluronic acid (HA) derivatives that have been developed for this purpose.

**Table 1:** 

Access this article online	
**Quick Response Code**:	**Website**: www.burnstrauma.com
	**DOI**: 10.4103/2321-3868.142398

Here in this monograph a narrative describing the application of the HA ester technology for the development of treatments for acute lesions, specifically burns, and the correspondent clinical results is presented.

Acute lesions are those which are not associated to a healing defect and that usually occur secondarily to a surgery or a trauma in a healthy individual. In these cases, the lesion may be deep and cause injury to muscles, blood vessels, nerves, bones and involve other body parts as well. It may also lead to severe loss of blood and tissues. Deep wounds that completely destroy the epidermis and part of the dermis have functional impacts as well, such as the impairment of skin oxygenation and effects on the tissue healing ability. Consequences can be very severe in terms of fluid management with the possibility of leading to dehydration and shock. Protein loss can also be serious, as well as the risk of contracting infections. To avoid all these dramatic consequences, prompt wound covering is an essential measure to be taken.[1] Standard clinical procedure comprises early escharectomy, followed by immediate tissue reconstruction with a skin graft.[2,3] This protocol represents the optimal solution for decreasing the risks related to wound infection, scar formation and excessive fluid loss and also allows the reduction of patient hospitalization time. In this light, the gold standard is represented by the use of autologous skin to cover the injury; nevertheless an immediate coverage with autologous skin in deep and extensive burns is not always possible. In these cases, alternative solutions have to be taken into considerations. For example, the grafting of cryopreserved, whole cadaver skin is a possible option to consider when autograft is not feasible, but problems related to availability and risk of viral transmission represent limitations for this technique.

Therefore, synthetic alternatives like dermal substitutes and advanced wound dressings have been designed and proposed for specific clinical use in burns.

Dermal substitutes are generally preferred in full-thickness lesions (such as traumas, surgical wounds, chronic ulcers, etc.) and have been shown to minimize hypertrophic scarring, contractures and increase scar elasticity in acute burn wounds.

On the other side, for the treatment of first and superficial second degree burns, affecting the epidermis and the superficial dermis, advanced wound dressing have been realized in order to promote wound healing by facilitating cell migration and proliferation and growth factor release.[4–6]

A variety of biomaterials for medical applications has been designed and developed in recent years and large efforts have been dedicated to the development of new biocompatible and biodegradable polymers which should release safe degradation products that enter into the normal metabolic pathway. Many advanced wound dressings consist of biomaterials made from various components of the extracellular matrix (ECM) and are theorized to favor healing by providing a structural scaffold and the signals important to complex cellular interactions during the healing phases. Among a series of possible materials, HA chemistry represents an interesting and valuable option for the development of medical devices for epidermal and dermal wound treatment.

## Hyaluronic acid based materials

The scientific rationale for the development of biomaterials based on HA relies on the characteristics of the material, which is a structural component of the ECM architecture and has an important role in water homeostasis that could favor tissue hydration, which is conducive to wound healing.

HA is an important polysaccharide components of the ECM and it is also plentiful in embryonic mesenchymal tissues. It is among the most hygroscopic natural molecules; when absorbing water, HA molecules can swell in volume up to 1000 times, forming loose hydrated matrices.[7] Probably, this character is the main cause behind many of its biological properties. The viscoelasticity of HA provides lubricating properties and shock-absorbing properties, that make it essential for the synovial fluid and the vitreous humor in the eye. Because of their high hygroscopicity, the solutions of HA are extremely osmotic, which is more significant in the presence of serum albumin, as is the case in most tissue fluids. In skin tissues, this is a key feature in regulating tissue hydration, especially in changing periods such as embryonic development, or during inflammatory processes after tissue damage, when the levels of HA are increased. Hyaluronan, or its acid form HA, is a polymer of disaccharides composed of D-glucuronic acid and D-N-acetylglucosamine, linked via alternating β-1,4 and β-1,3 glycosidic bonds. Hyaluronan can have up to 25,000 disaccharide repeat units in length and range in size approximately from 5,000–20,000,000 Da *in vivo*. Depending on the different molecular weights of the HA molecule, HA has been observed to play different roles in the body. Considering exogenous HA, the high molecular weight properties are related to angiogenesis inhibition and to a mostly physical role, while low molecular weight HA has been associated with a modulating effect on the inflammatory process due to its action on free radicals,[8–10] its antioxidant action[11] and by excluding lithic enzymes from the immediate cellular environment and other extracellular matrix components.[8,12] This moderating action can contribute to the stabilization of granulation tissue matrix, which is rich in HA.[13,14] In fact, even if inflammation is part of granulation tissue formation, it needs to be modulated in the normal tissue healing process. The process by which HA fragments promote granulation tissue formation is complicated and has been associated to superior collagen deposition.[15]

Endogenous HA plays several architectural roles in the extra cellular matrix (ECM) organization by binding with cells and other components through specific and non-specific links. HA specifically interacts with several proteoglycans (such as aggrecan, versican) through HA-binding motifs and organizes the networks of fibrin, fibronectin and collagen.[7]

The HA molecule has been shown to contribute as a modulator of many of the biological processes thanks both to its physicochemical and biological characteristics. For example, it has been reported that high tissue concentrations of HA are present during the activation of key biological processes such as remodelling, regeneration and morphogenesis.

Interestingly, HA is involved in the early stages of tissue repair and wound healing being, together with fibrin, a component of the matrix which forms to aid fibroblast and endothelial cell organization into the injury site. Specifically, HA hydrophilic properties make the fibrin clot softer and easier for the cells to colonize. These cellular processes are essential steps to allow progress of the tissue regeneration process as they allow the structural construction of the newly-forming tissue.[16,17]

Additionally, HA plays a definite role in controlling the angiogenic process. This modulation role is driven by the molecular weight. In fact, it has been demonstrated that high molecular weight-HA is an inhibitor of angiogenesis[18] and exerts a predominantly architectural role while, on the other hand, the oligosaccharides of low molecular weight-HA have shown a marked angiogenic effect in a series of experimental models,[19–21] besides stimulating collagen production in endothelial cells.[22]

In order to develop appropriate regenerative medicine strategies, fetal wound healing has been studied as an ideal tissue healing condition as it is characterized by the lack of fibrotic scar formation. Remarkably, a prolonged presence of HA has been reported to be associated to the scarless fetal tissue repair,[23–26] and in relation to this observation, some authors proposed that a HA rich environment inhibits the matrix cells responsible for scar formation.[27,28]

Considering the specific issue of skin wound and healing, the use of HA as starting polymer to develop a medical material is suggested by the role that the molecule plays in normal epidermis, where it is found in relatively high concentration in the basal layer.[29,30] In this tissue HA main functions are related to the extracellular space 3-dimensional architecture, to the structure hydration for nutrient flow and to the involvement in keratinocyte proliferation and migration.[31] For all of the above described reasons, exogenous HA has been investigated in wound healing applications and the results coming from those studies demonstrated that HA is effective even in challenging conditions such as chronic wounds. *In vivo* preliminary experiments designed to characterize HA’s role in healing of different tissues demonstrate that topically applied HA is able to favor skin wound healing in rats[32,33] and hamsters[34] and perforated tympanic membrane healing in rats.[35]

Nevertheless, poor physical properties of this natural polymer (that is short residence time *in vivo* and solubility) represent a serious limitation for its direct use in the medical field.[36] Methods of chemical modification of natural polymers have been applied to HA to produce a material with enhanced resistance to degradation and therefore longer residence time and physical and chemical properties that allows for multiple manufacturing processes.[37]

### Clinical experience with hyaluronic acid based dressings to treat burns

HYAFF® 11 is a HA derivative polymer obtained by the esterification of the free carboxylic group of glucuronic acid with benzylic alcohol with the aim of improving the polymer stability, while maintaining the biological and safety characteristics of the starting molecule.[38] This chemical modification decreases the hydrophilic carboxyl groups and increases the hydrophobic components of the polysaccharide. Upon esterification, the obtained biopolymer can be processed to manufacture materials with several types of physical conformations, therefore suitable for a wide range of clinical applications. The chemical process allows control over the percentage of esterification, which has found to be correlated to solubility properties. HYAFF® 11 degradation has been studied and described through dedicated experiments and it has been observed that the material is undergoing hydrolytic degradation of the ester bond, conducting to a gradual de-esterification of the polymer which is then increasingly hydrated, becoming more similar to native HA.[38] *In vivo* data have confirmed the biocompatibility of the biomaterial and its safe degradation through a well-known metabolic pathway.

Recently, the use of different HA derivatives in healing burns and epithelial surgical and chronic wounds has been evaluated with a systematic review of the literature and a meta-analysis taking into account relevant randomized controlled trials. A positive effect of HA in the healing of chronic wound ulcers of various etiologies, burns and epithelial surgical wounds was observed no matter the form in which HA is delivered, showing that HA derivatives favor the healing process.[39] The results of the analysis performed additionally pointed out that HA derivatives significantly improved the healing of wounds versus traditional standards of care.

Hyalomatrix and Hyalosafe are HA based medical devices dedicated to wound healing that have been designed, developed and manufactured using HYAFF® 11 technology. During their design and development process, the main requirements considered were the need of providing a temporary coverage for the wound via an off-the-shelf approach, the possibility of facilitating the healing process and specifically the dermal regeneration and biological safety of the overall approach. Moreover the ease of use and affordability were also considered as important points.

### HA based dermal substitute use in burn treatment

**Figure 1:**
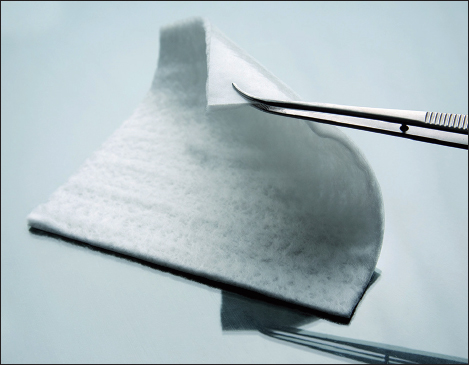
Hyalomatrix is a bi-layered dermal substitute. In this picture the HYAFF® 11 layer is in the front, while the back side is the silicone layer.

Hyalomatrix is a bi-layered, sterile, flexible and conformable dermal substitute designed to provide immediate wound closure and to promote the permanent regeneration of dermis [Figure 1]. The layer in contact with the wound is a 3D fibrous matrix made of HYAFF® 11, while the top layer is a thin and transparent silicone membrane. The HYAFF® fibrous matrix layer, once in contact with the wound bed, integrates and starts the release of HA. This biodegradable matrix acts as a scaffold for cellular invasion, dermal matrix component deposition and organization and capillary growth, while stimulating dermal (fibroblasts) and vascular (endothelial) cells organization at the wound site.[2,40–42] Following spontaneous degradation through the de-esterification process, the HYAFF® fibers release HA in a prolonged manner,[41] which favors epidermal cells migration allowing a spontaneous re-epithelization from the wound margins. These actions lead to new dermal tissue reconstruction and, in many cases, to spontaneous re-epithelization. On the outer side, the thin silicone layer protects the wound from external contaminations and prevents excessive fluid loss. Moreover, being transparent, it allows continuous monitoring of the wound healing process without dressing removal. The silicone layer is designed to be easily removed without pain or wound bed damage following the formation of the neo-tissue.

Hyalomatrix is currently indicated for clinical use as a dermal substitute for the immediate coverage, following surgical excision and prior to grafting, of deep burns and partial to full-thickness post-traumatic, post-surgical or deep-chronic wounds, such as vascular ulcers and diabetic foot ulcers. In recent years, clinical evidence has been collected and published to report the result of the treatment with Hyalomatrix in this application field.

Subsequently Gravante *et al.* and Osti, published larger clinical experiences on the use of Hyalomatrix in acute wounds such as burns.[43,44]

A multicenter retrospective national survey (2005–2006) involving 11 burn care centers, 57 patients, with deep partial (*n* = 31, 54.4%) and full thickness burns (*n* = 22, 38.6%) was performed. Mean follow-up was 37 days (range 1–472 days). Cleansing/debridement was conducted in 42.1% of cases (*n* = 24), dermabrasion in 28.1% (*n* = 16), escharectomy in 26.3% (*n* = 15), then Hyalomatrix was applied. A total of 53.6% (30/56) of cases underwent grafting.[43]

On day 7 after the use of Hyalomatrix, reepithelization was observed more frequently in deep partial thickness than in full thickness burns, while no significant differences between adults and young patients were reported. After 29 days (second evaluation), almost all patients were present and complete closure was achieved in 32.7% of patients. At the last evaluation, complete closure of the wound was achieved in 85.7% of presented patients (28/57), with 14.3% (*n* = 4) still having a partial reepithelization. No differences were observed for burn depth or patients undergoing grafting, while the Vancouver Scar Scale showed better values for adults. Receiving graft is more frequent in adults (69.6%) than young patients (47.4%). Thus, Hyalomatrix has been applied to young and adults, in deep partial and full thickness burns, either temporarily as wound coverage before grafting or alone for wound healing.[43] No adverse reactions have been reported. The study outcomes demonstrate that Hyalomatrix is good in safety profile, and effective as a skin regenerative matrix.

The skin pH variation from the acute phase to the epithelialization was observed in a burn patient that was first medicated with a hydrogel, Burnshield (Levtrade International (Pty) Ltd) and then treated with Hyalomatrix until complete epithelialization.[44] The pH was measured from day 1 post-burn and every other day, thereafter until complete re-epithelialization. Alkaline pH values were found for the burned skin from the day of the burn until day 12, with an alkaline pH peak on day 4; the values then gradually returned to normal (pH, 5.5) from day 13 onwards.

As pediatric patients are a large and significant population in burn treatment, specific studies were carried out to evaluate the Hyalomatrix administration in this group.

With specific reference to pediatric patients, a study on 300 subjects was performed using Hyalomatrix as a temporary dermal substitute to cover deep partial-thickness burns after dermabrasion.[45,46] The dermabrasion technique followed by coverage of the treated area, in fact, did improve the prognosis of burns victims and the correspondent aesthetic and functional outcomes, especially in the pediatric patient population.[46,47] This intermediate step with Hyalomatrix treatment was introduced in order to remove necrotic debris and to stimulate tissue regeneration in a wet and protected environment. In this study, 83% of patients had healing within day 21, indicating that the application of Hyalomatrix after dermabrasion is a useful and feasible approach for the treatment of deep partial-thickness burns. Some kind of hypertrophic processes were observed in almost all patients during the first year, however in 90% of the cases, hypertrophic scars disappeared within 1 year, and in 96% in 2 years. Only 4% of cases (*n* = 12) had hypertrophic scars that lasted for more than 2 years requiring treatment with intralesional corticosteroids injections. Additionally, the incidence of infection was investigated in comparison to the historical data within the same center, indicating a decrease of their occurrence to 10%, as compared to the historical results of 29.5%.

An additional experience with the clinical use of Hyalomatrix was reported in the case of tendon exposure and loss of substance following burns, trauma or chronic injuries.[48] In the study, 30 patients underwent surgical debridement followed by autologous platelet-rich plasma (PRP) gel injection. PRP may be injected intralesionally, intratendonally or perilesionally, or mixed with autologous thrombin in a 9:1 ratio forming a platelet gel; the wounds were then covered with Hyalomatrix. The severity of open wounds was noticeably reduced after treatment with PRP and the HA matrix — only one patient needed a free flap to cover exposed bone and tendon. Based on these results and the ones from previous studies, it is suggested that using PRP and the HA matrix together significantly enhances the formation of granulation tissue and helps prevent infection.[49] A series of 10 cases was also reported in which loss of substance due to trauma, tumor or third-deep burns (*n* = 5) was treated with Hyalomatrix, followed by a thin skin graft.[50] Good efficacy was reported in the short term and at 12 months. A flexible skin equivalent to that observed with collagen acellular dermal substitute as well as lack of adhesion of the tendon graft skin was observed which confirmed the dermal regeneration.

In summary, Hyalomatrix is a bilayered device, comprised of a wound contacting scaffold-like material and a semi-permeable silicone membrane. It is an advanced wound dressing that can be readily used as a skin substitute for wound coverage, following the surgical preparation of the wound and before grafting in burn patients. In second degree burns, after the removal of necrotic tissues, it can be used to protect the residual dermal layer and may also stimulate the reepithelization from the wound edge and skin appendages. Contrarily, in full thickness burns and cases that can’t undergo skin grafting immediately, Hyalomatrix can be used as a temporarily treatment after the wound cleaning for wound bed protection prior to the definitive treatment of skin grafts or other procedures such as cultured autologous keratinocytes.

### HA film wound dressing in burn treatment

**Figure 2:**
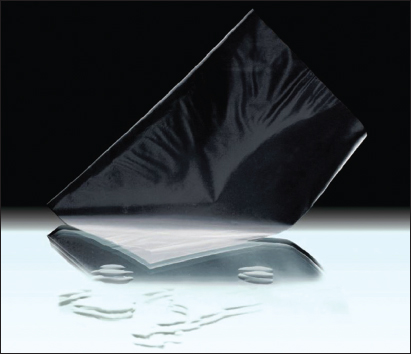
Hyalosafe is a transparent film wound dressing.

Using the same HYAFF® 11 technology, another product, named Hyalosafe, was designed and developed to provide effective coverage for superficial wounds. Hyalosafe is a transparent film wound dressing [Figure 2] indicated for use in the treatment of first and superficial second-degree burns. The use of the product in deep partial thickness burns in pediatric patients to cover the burn at recovery, prior to dermabrasion has also been reported.[51]

The HYAFF® 11 film is applied directly on the lesion, keeping the wound moist and thus creating the ideal conditions for a rapid re-epithelialization, avoiding the risk of tissue maceration. Thanks to the release of hyaluronic acid during HYAFF® 11 degradation, Hyalosafe interacts with the lesion environment, facilitating the renewal of the epithelium. Importantly, as this membrane does not adhere to the wound, its removal is painless for the patient.

The use of HYAFF® 11 film in the treatment of second-degree face burns[52] has been reported on 40 children. Face burns are an important problem which often requires hospitalization and represent 25% of the overall extensive pediatric burns. This type of burns is related to an important fluid loss due to the characteristics of the affected tissue. In general, epidermal and superficial dermal wound have the ability to recover without scarring in absence of infections, therefore there is a strong need for treatments that are able to protect the burned area. Hyalosafe was used for this purpose showing very good healing properties and leading to notable aesthetic results. No case of wound infection was reported.

## Conclusion

In conclusion, important tools based on HA biopolymers are now available for the treatment of acute wounds, such as burns. These biomaterials have been designed and developed to realize advanced dressings able to protect the wound and facilitate healing combining the properties of HA and the improved characteristics of the ester derivatives which assure long term residence time and processibility.

This class of devices represents a valuable clinical option for burn management as confirmed by the current clinical experience with Hyalomatrix and Hyalosafe.
